# Three-Dimensional Iron Oxide Nanoparticle-Based Contrast-Enhanced Magnetic Resonance Imaging for Characterization of Cerebral Arteriogenesis in the Mouse Neocortex

**DOI:** 10.3389/fnins.2021.756577

**Published:** 2021-11-26

**Authors:** Till de Bortoli, Philipp Boehm-Sturm, Stefan P. Koch, Melina Nieminen-Kelhä, Lars Wessels, Susanne Mueller, Giovanna D. Ielacqua, Jan Klohs, Peter Vajkoczy, Nils Hecht

**Affiliations:** ^1^Department of Neurosurgery, Charité-Universitätsmedizin Berlin, Corporate Member of Freie Universität Berlin, Humboldt-Universität zu Berlin, Berlin, Germany; ^2^Center for Stroke Research Berlin (CSB), Berlin, Germany; ^3^Department of Experimental Neurology, Charité-Universitätsmedizin Berlin, Corporate Member of Freie Universität Berlin, Humboldt-Universität zu Berlin, Berlin, Germany; ^4^NeuroCure Cluster of Excellence and Charité Core Facility 7T Experimental MRIs, Charité-Universitätsmedizin Berlin, Corporate Member of Freie Universität Berlin, Humboldt-Universität zu Berlin, Berlin, Germany; ^5^Institute for Biomedical Engineering, University of Zurich and ETH Zürich, Zurich, Switzerland

**Keywords:** contrast-enhanced MRI, cerebral blood volume, iron oxide nanoparticles, stroke, cerebrovascular disease, vessel imaging

## Abstract

**Purpose:** Subsurface blood vessels in the cerebral cortex have been identified as a bottleneck in cerebral perfusion with the potential for collateral remodeling. However, valid techniques for non-invasive, longitudinal characterization of neocortical microvessels are still lacking. In this study, we validated contrast-enhanced magnetic resonance imaging (CE-MRI) for *in vivo* characterization of vascular changes in a model of spontaneous collateral outgrowth following chronic cerebral hypoperfusion.

**Methods:** C57BL/6J mice were randomly assigned to unilateral internal carotid artery occlusion or sham surgery and after 21 days, CE-MRI based on T2*-weighted imaging was performed using ultra-small superparamagnetic iron oxide nanoparticles to obtain subtraction angiographies and steady-state cerebral blood volume (ss-CBV) maps. First pass dynamic susceptibility contrast MRI (DSC-MRI) was performed for internal validation of ss-CBV. Further validation at the histological level was provided by *ex vivo* serial two-photon tomography (STP).

**Results:** Qualitatively, an increase in vessel density was observed on CE-MRI subtraction angiographies following occlusion; however, a quantitative vessel tracing analysis was prone to errors in our model. Measurements of ss-CBV reliably identified an increase in cortical vasculature, validated by DSC-MRI and STP.

**Conclusion:** Iron oxide nanoparticle-based ss-CBV serves as a robust, non-invasive imaging surrogate marker for neocortical vessels, with the potential to reduce and refine preclinical models targeting the development and outgrowth of cerebral collateralization.

## Introduction

Cerebral collateral remodeling (cerebral arteriogenesis) represents an endogenous rescue mechanism to cope with chronic cerebral hypoperfusion and determines the severity and outcome of hemodynamic stroke (Brozici et al., 2003; Todo et al., 2008; Menon et al., 2013; Lima et al., 2014). Despite this critical importance of collateral arteries, their clinical and neuroradiological assessment remains challenging because standard neuroimaging methods to study collateral circulation are hampered by a low spatial resolution (Shuaib et al., 2011; Campbell et al., 2013). High spatial detail is important because next to the basal and leptomeningeal vasculature (Buschmann et al., 2003; Kitagawa et al., 2005; Schneider et al., 2007), arteriolar remodeling also occurs at the level of the subsurface resistance vessels (Hecht et al., 2012, 2015; Marushima et al., 2019) that represent a bottleneck to cortical perfusion (Nishimura et al., 2007).

For high-resolution imaging of the cerebral vasculature, contrast enhanced magnetic resonance imaging (CE-MRI) with ultrasmall, carbohydrate-coated superparamagnetic iron oxide nanoparticles and gradient echo or susceptibility weighted imaging has previously been reported (Iv et al., 2018; Buch et al., 2021). The resulting contrast to noise ratios are generally higher for these particles due to an exceptionally high transverse relaxivity compared to conventional gadolinium chelates based contrast agents (Rohrer et al., 2005; Plock et al., 2015; Knobloch et al., 2018). Although high-resolution angiographies are technically feasible in humans (Liu et al., 2018), animal models are needed for validation of such techniques to allow reliable, dynamic assessment of cerebrovascular development and remodeling in health and disease. Thus far, different approaches have been described to quantify iron oxide nanoparticle-based CE-MRI: On the one hand, blood volume fraction estimated by steady state cerebral blood volume (ss-CBV) measurements using a biophysical model in the static dephasing regime may serve as an *indirect* marker of gross total blood vessel volume (Boxerman et al., 1995). Technically, ss-CBV benefits from the long circulating half-time of iron oxide nanoparticles, which permits high-resolution assessment of perfused vasculature in the context of MRI (Plock et al., 2015). On the other hand, contrast enhanced magnetic resonance *angiography* (CE-MRA) was shown to allow *direct* detection of small-caliber parenchymal blood vessels in the mouse cerebral cortex using a semi-automated vessel tracing algorithm that permits diameter quantification among blood vessels enhanced at steady-state (Klohs et al., 2012). These findings are highly relevant, because non-invasive, longitudinal characterization of the cerebral vasculature has immediate implications for reduction and refinement of animal experiments next to understanding the etiology and course of neurovascular and neurodegenerative diseases. The current dilemma is that the reliability of both indirect but also direct CE-MRI techniques remains unclear, because a systematic validation against the actual cerebral angioarchitecture has not yet been performed. Therefore, the aim of the present study was to validate iron oxide nanoparticle-based CE-MRI for blood vessel quantification in a preclinical mouse model of chronic cerebral hypoperfusion and spontaneous collateral remodeling by testing a previously reported vessel tracing algorithm and analyzing steady state blood volume maps (ss-CBV) against serial two-photon (STP) tomography.

## Materials and Methods

### Ethics Statement

Experiments were permitted by the local ethics committee on animal research (Landesamt für Gesundheit und Soziales, Berlin, Germany; LAGeSo G0186/16) and in conformity with the German Law for Animal Protection and the National Institute of Health Guidelines for Care and Use of Laboratory Animals. Experiments were reported following the ARRIVE guidelines.

### Animals and Surgery

Sixteen male C57BL/6J mice aged 12 weeks and an initial body weight ranging from 27 to 32 g (Charles River WIGA GmbH, Sulzfeld, Germany) were randomly assigned to permanent unilateral internal carotid artery occlusion (ICAO) for stimulation of spontaneous cerebral arteriogenesis (*n* = 8) or sham surgery (Sham) (*n* = 8), as previously described (Hecht et al., 2012). Briefly, mice were anesthetized with 70 mg/kg ketamine and 16 mg/kg xylazine and body temperature was maintained at 37°C. Animals were positioned supine and the right-sided ICA was exposed by a midline neck incision. For ICAO, the right ICA was permanently ligated with 8/0 silk. For Sham, the ICA was exposed but no ligation was performed. The skin was sutured with 6/0 nylon. Mice were kept in an enriched environment with free access to food and water.

### Magnetic Resonance Imaging

On Day 21 after surgery, a venous catheter was placed into the femoral vein and CE-MRI was acquired at 7 Tesla with a small animal MR scanner (Bruker BioSpin GmbH, Ettlingen, Germany) using a transmit/receive mouse head cryoprobe (Bruker BioSpin GmbH, Ettlingen, Germany). Mice weight at Day 21 was 34.1 ± 9.7 g for ICAO and 30.2 ± 7.2 g for Sham. Researchers performing the CE-MRI were blinded to the group assignment. The MRI protocol included the following sequences:

• T2-weighted MRI [2D Rapid Acquisition with Relaxation Enhancement (RARE), 39 contiguous 0.4 mm-thick slices, field of view (FOV) = (18.72 mm)^2^, (80 μm)^2^ in plane resolution, repetition time/effective echo time (TR/TE) = 4.2s/33 ms, echo spacing 11 ms, RARE factor 8, bandwidth (BW) = 32.9 kHz, 2 averages, acquisition time (TA) = 4:03 min].
• CE-MRA of the cortex by acquiring T2*-weighted images [3D fast-low-angle-shot, FOV = 13.52 mm^3^ × 16.64 mm^3^ × 5.2 mm^3^, (65 μm)^3^ resolution, TR/TE = 54 ms/3.9 ms, FA = 20°, BW = 50 kHz, partial Fourier imaging (PFT) in frequency encoding direction with factor 1.33, TA = 29:57 min per image] first pre-injection and then post-injection of ultrasmall, superparamagnetic iron oxide nanoparticles (Ferumoxytol, AMAG Pharmaceuticals, Waltham, MA) through the femoral vein. Iron oxide nanoparticles (0.300 mmol/l) were diluted 1:3 (volume/volume) in 0.9% sodium chloride and 1.6753 μl per gram bodyweight of the diluted contrast agent was injected as a single bolus over 2–3 s, which yielded a final contrast agent concentration of 300 μmol Fe/kg bodyweight.
• Dynamic susceptibility contrast (DSC)-MRI during iron oxide nanoparticle injection [single shot gradient-echo echo-planar imaging, 15 contiguous 0.4 mm slices, FOV = 18.72 × 18.72 mm^2^, (293 μm)^2^ resolution, TR/TE = 400 ms/7.2 ms, FA = 50°, BW = 200 kHz, PFT = 1.5 in phase encoding direction, 200 repetitions, TA = 1:20 min].

T2-weighted sequences and DSC-MRI FOV covered the whole brain, whereas the CE-MRA FOV only covered the dorsal neocortex. A set of 5 saturation slices with identical geometry was used to suppress aliasing artifacts originating from the signal of the ventral part of the brain.

### Blood Vessel Staining

To visualize cerebral blood vessels for histological analysis, mice received an *in vivo* injection of 100 μl fluorescein-labeled Lectin [DyLight 488 Labeled Lycopersicon Esculentum (Tomato) Lectin, DL-1174, Vector Laboratories, Burlingame, CA] though the femoral vein to stain endothelial cells of perfused blood vessels. Next, the animals were transcardially perfused with 4% paraformaldehyde in phosphate buffered saline and the isolated brains were stored in the same solution at 4°C for 72 h and then kept in 0.1% sodium azide in phosphate buffered saline.

### Histology

For the purpose of 3R and resource conservation during histological analysis, animals were randomly assigned to two groups: In the first group, *n* = 4 ICAO and *n* = 4 Sham animals were used to validate our animal model and confirm neocortical vascular remodeling on Day 21 after unilateral ICAO by confocal laser scanning microscopy (Leica TCS SP8, Leica Camera AG, Wetzlar, Germany) of whole-mount brain sections of 80 μm thickness with 1.5 × 1.5 × 8.9 μm^3^ [X × Y (plane) × Z (depth)] voxel resolution and laser excitation at 488 nm. The resulting confocal cerebral blood vessel volume (CBV) was defined as confocal-CBV by the fraction of Lectin-positive voxels from all tissue voxels in the right-sided neocortex (Supplementary Figures 1, 2).

After neocortical vessel outgrowth following ICAO was confirmed, *n* = 2 ICAO and *n* = 1 Sham animals were used in the second group to validate our iron oxide nanoparticle-based ss-CBV method at whole brain level by using serial two-photon tomography (STP) of perfused brains that was generated with a commercially available platform (TissueVision, Newton, MA, United States) (Ragan et al., 2012). Briefly, block-face 2-photon imaging of brains was performed by slicing 50 μm sections with measurements of two 25 μm optical planes per slice and three channels (RGB) leading to a final resolution of 1.2 μm × 1.2 μm in plane and 25 μm slice thickness. The resulting serial two-photon cerebral blood volume was defined as STP-CBV.

### Image Processing

Image co-registration was used to compare MRI and histological data and to annotate brain regions from the Allen brain atlas. MR image pre-processing was performed in ImageJ (v1.52e)^1^ according to the following steps: First, all images were transformed to the resolution of the CE-MRI scan using nearest-neighbor interpolation. Second, fixed relations in image geometry planning during acquisition were used to transform images to a final FOV = 18.72 mm^3^ × 18.72 mm^3^ × 15.2 mm^3^, (65 μm)^3^ isotropic resolution and matching geometry without image registration techniques. Manual correction of shifts due to scanner imperfections were corrected in steps of one voxel using a custom ImageJ plugin and 3 degrees of freedom and nearest neighbor interpolation. T2-weighted images were warped to the Allen brain atlas using the MATLAB (v. R2014a, MathWorks, Natick, MA, United States) toolbox ANTx (Koch et al., 2019). The calculated registration was then applied to all other MRI measurements. Histology images were registered to the Allen brain atlas using custom MATLAB tools (R2016a, The MathWorks, Inc., Natick, MA, United States) (Koch et al., 2019). The following steps were performed to transform STP data to the Allen brain atlas:

• Background removal by filtering the sum of RGB images (2D Gaussian filter, standard deviation of 11) on the first of the optical planes (resulting mask was applied to the second).
• Generation of (A) an anatomical image for later image registration from autofluorescence images in the red channel and (B) a vessel image by feeding the RGB-channel images into a principal component analysis and extracting the 2nd principal component.
• Automated identification of vasculature on the vessel image by closing gaps with 3 × 3 pixel kernel, removing salt and pepper noise with 2D median filter with a 9 × 9 kernel, clustering into spatially connected objects removing objects with <5 connected pixels and binarization (values > 0 set to 1).
• Down-sampling of the binarized vessel image to the resolution of the Allen template (voxel size: 0.025 μm^3^ × 0.025 μm^3^ × 0.025 μm^3^) using bilinear interpolation. This led to an image containing values between 0 and 1 representing the fraction of 1s of the high-resolution vessel image in the low-resolution voxel, i.e., a map of STP-CBV. This map and the down-sampled template-like image were saved as nifti-files.
• Registration of STP-CBV map via template-like image (moving image) onto the Allen mouse template (fixed image) in Elastix using a rigid body transformation, followed by affine transformation and a subsequent B-spline transformation (Klein et al., 2010; Shamonin et al., 2013).

### Vessel Tracing Analysis Based on Contrast Enhanced Magnetic Resonance Angiography

Angiograms from CE-MRI (=CE-MRA) were generated by subtraction of image intensities S_*pre*_ – S_*post*_. From these, vessel densities were calculated by a vessel tracing algorithm as described previously (Klohs et al., 2012). Based on previous findings in our model and as suggested by the results from our confocal-CBV analysis (Supplementary Figures 1, 2), we hypothesized an increase in vessel densities in the right-sided neocortex of ICAO animals compared to Sham (Hecht et al., 2012, 2015; Marushima et al., 2019).

### Steady State Cerebral Blood Volume Analysis From Iron Oxide Nanoparticle-Based Contrast-Enhanced Magnetic Resonance Imaging

The change in transverse relaxation rate ΔR_2_* = 1/TE × ln (S_*pre*/_S_*post*_) was calculated using ImageJ and ss-CBV was defined using the biophysical model of Yablonsky and Haacke via ss-CBV = 3/(4πγΔχB_0_)ΔR_2_* with the gyromagnetic ratio γ = 2.67502 × 10^8^ rad/s/T and susceptibility difference Δχ = 0.282 × 10^–8^ (CGS units) between blood and tissue at B_0_ = 7 T (Lemasson et al., 2016) assuming linear increase with contrast agent dose and saturation of iron magnetization above 4.7 T (Yablonskiy and Haacke, 1994; Lemasson et al., 2015).

### Dynamic Susceptibility Contrast Relative Cerebral Blood Volume Analysis From Iron Oxide Nanoparticle-Based Dynamic Susceptibility Contrast Magnetic Resonance Imaging

Dynamic susceptibility contrast relative cerebral blood volume was calculated using DSCoMAN Version 1.0 (Duke University Medical Center, Durham, NC, United States) (Boxerman et al., 2006). Using ImageJ, raw DSC-MRI images were manually converted into a stack with xyztc order, one channel, 15 slices, and 200 frames. Pixel width and height were set to 0.2925 mm, voxel depth to 0.4 mm. The frame interval was set to 0.4 s, the origin to 0.0 pixels. A timetable was imported. Next, the DSCoMAN GUI was started within ImageJ to calculate raw perfusion images. The first 20 timepoints were excluded for the analysis of rCBV. Last baseline time, last time, slice locations, time points, echo time (msec), whole brain threshold, noise SDs, as well as parameter for last baseline time computation were not modified from the values automatically given by DSCoMAN. The resulting rCBV map was scaled as the raw DSC-MRI images above and saved as a nifti file.

### Statistical Analyses and Methods to Prevent Bias

Since no data on CE-MRI was available for our mouse model, we chose an exploratory study design with similar sample size to the original study describing the mouse model with histological methods (Hecht et al., 2012). A total of 16 mice underwent MRI measurements. The researchers performing the MRI measurements were blinded to the group assignment. One animal was excluded because of clots in the brain after iron oxide nanoparticle injection, one animal died during the MRI protocol leading to the final group size of 7 per group. All analysis was performed blinded to group assignment. A volume-of-interest of the right neocortex for vessel tracing was defined manually by a blinded researcher and no iterations were made in the analysis. The right-sided neocortex defined by the Allen Mouse Brain Atlas was chosen as volume-of-interest for ss-CBV and DSC-rCBV analysis prior to the analysis (Supplementary Figure 3; Lein et al., 2007). Analysis of volume-of-interests in processed images was performed using custom code. Plots and statistics were performed using R version 4.0. Individual data points were visualized together with a summarized mean value ± standard deviation. Data were statistically described by the *p*-value derived from 2-sided Student’s t-tests after passing a Shapiro-Wilk normality test. Spearman’s rank correlation coefficient (*r*) was used to describe the correlation between ss-CBV and h-CBV.

## Results

### Assessment of Vessel Density With Semi-Automated Blood Vessel Tracing Algorithm

**FIGURE 1:**
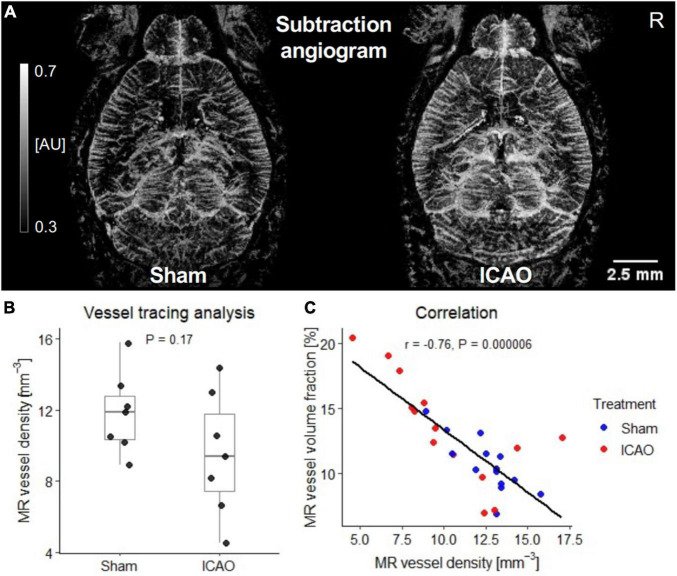
Quantitative contrast enhanced magnetic resonance angiography (CE-MRA) subtraction angiogram analysis 21 days after ICAO or Sham. **(A)** Iron oxide nanoparticle-based MR subtraction angiograms in gray-scaled arbitrary units (AU) clearly visualized a stronger enhancement across both hemispheres on Day 21 after ICAO compared to Sham. **(B)** In contrast to this clearly visualized effect, semi-automated MR vessel density analysis yielded a lower instead of higher vessel density in the right neocortex after ICAO (*n* = 7 per group). **(C)** Relationship between MR vessel density and the corresponding vessel volume fraction in neocortex measurements from both hemispheres described by Spearman’s rank correlation coefficient (*n* = 28).

Blood vessels in the right neocortex 21 days after ICAO or sham surgery were precisely visualized in a three-dimensional space after subtraction of post-contrast from pre-contrast images. As expected from previous findings^11^ and our confocal modal validation (Supplementary Figures 1, 2), more neocortical vessels were visible on CE-MRA subtraction angiograms 3 weeks after ICAO throughout both hemispheres (Figure 1A). Unexpectedly, however, vessel densities calculated by a previously described, semi-automated vessel tracing algorithm tended to be lower in ICAO than in Sham-treated animals (Sham: 11.8 ± 2.3 mm^–3^; ICAO: 9.5 ± 3.5 mm^–3^; *p* = 0.17; *n* = 7 per group; Figure 1B). Thus, although measurements within the ICAO group displayed large MR vessel volumes that visually corresponded well to the subtraction angiograms, the tracing analysis did not translate these effects into an increased MR vessel density. Additionally, we compared MR vessel densities for both groups and hemispheres with corresponding MR vessel volumes in a *post hoc* analysis and, interestingly, MR vessel densities were in strong negative correlation to MR vessel volumes (*r* = -0.76; *p* = 0.000006; *n* = 28; Figure 1C).

### Assessment of Vessel Density With Iron Oxide Nanoparticle-Based Steady State Cerebral Blood Volume and Dynamic Susceptibility Contrast-Relative Cerebral Blood Volume

**FIGURE 2:**
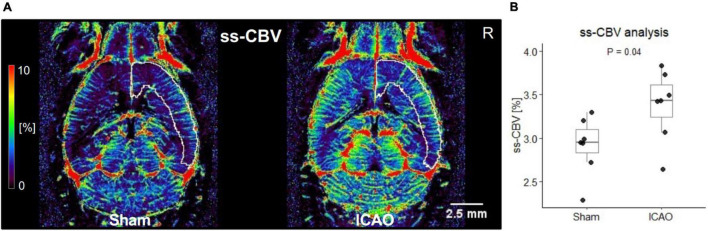
Steady state-cerebral blood volume (ss-CBV) analysis 21 days after ICAO or Sham. **(A)** Iron oxide nanoparticle-based ss-CBV maps color-coding the fraction of blood volume per voxel brain tissue in percent (%). The white frames illustrate the right-sided neocortex as defined by the Allen Mouse Brain Atlas. **(B)** Group comparison in the right-sided neocortex ipsilateral to the permanent ICA-occlusion shows an increase in ss-CBV after ICAO (*n* = 7 per group).

**FIGURE 3:**
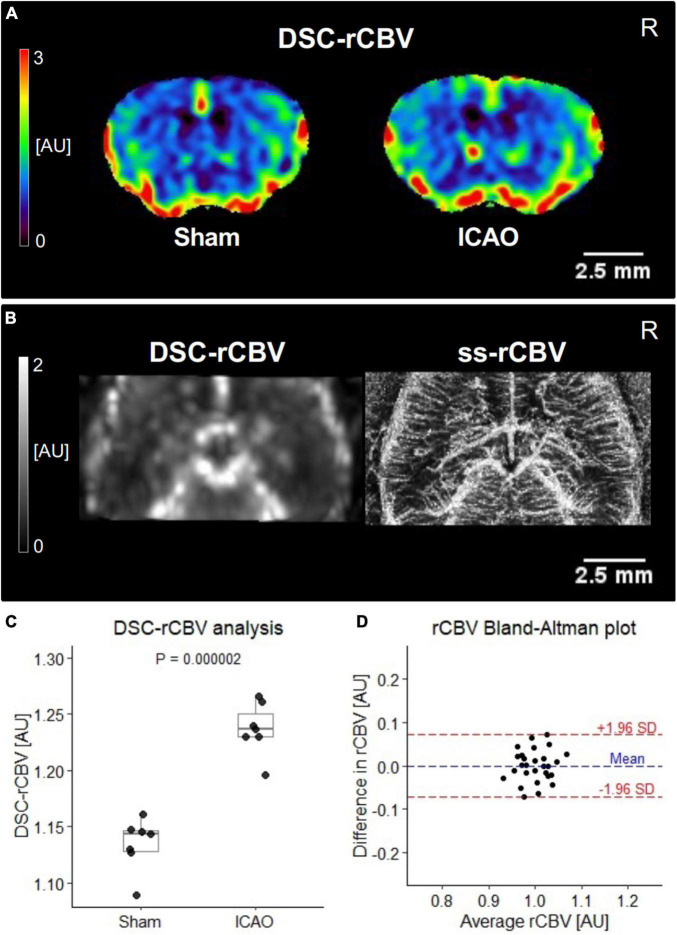
Internal cerebral blood volume (CBV) validation. **(A)** Color-coded normalized dynamic susceptibility contrast-relative cerebral blood volume (DSC-rCBV) maps in arbitrary units (AU). **(B)** Comparison of normalized DSC-rCBV and ss-CBV map resolution for the same animal. **(C)** Comparison of iron oxide nanoparticle-based DSC-rCBV at Day 21 (*n* = 7 per group). **(D)** Bland-Altman comparison of DSC-rCBV and ss-CBV in the right- and the left-sided neocortex (*n* = 28). The average of ss-CBV and DSC-rCBV is plotted against their difference. A mean difference of 0.000007 AU in rCBV with limits of agreement between -0.07 and 0.07 AU was noted.

Next, we explored whether ss-CBV maps could serve as a surrogate marker for parenchymal blood vessel imaging (Figure 2A) and found a significant increase of ss-CBV in the right neocortex following ICAO (Sham: 2.9 ± 0.3%; ICAO: 3.4 ± 0.4%; *p* = 0.04; *n* = 7 per group; Figure 2B). Cerebral hemodynamics were also assessed by DSC-MRI, which serves as a commonly applied technique for blood volume mapping by MRI. For between-subject comparisons of relative DSC-CBV (DSC-rCBV) in the parenchyma, the right hemisphere was normalized to the mean value of all measured voxels (Figure 3A). In agreement with our parenchymal ss-CBV analysis but at clearly lower spatial resolution (Figure 3B), a significant increase in DSC-rCBV following ICAO was noted for the right neocortex (Sham: 1.13 ± 0.02 AU; ICAO: 1.24 ± 0.02 AU; *p* = 0.000002; *n* = 7 per group; Figure 3C). To confirm the internal validity of our ss-CBV blood volume analysis, neocortical ss-CBV and DSC-rCBV measurements from both hemispheres were normalized and compared with each other using a Bland-Altman plot that yielded a mean difference of 0.000007 AU with limits of agreement of –0.07 to 0.07 AU; *n* = 14; Figure 3D).

### Histological Validation of Iron Oxide Nanoparticle-Based Steady State-Cerebral Blood Volume Against Serial Two-Photon Tomography

**FIGURE 4:**
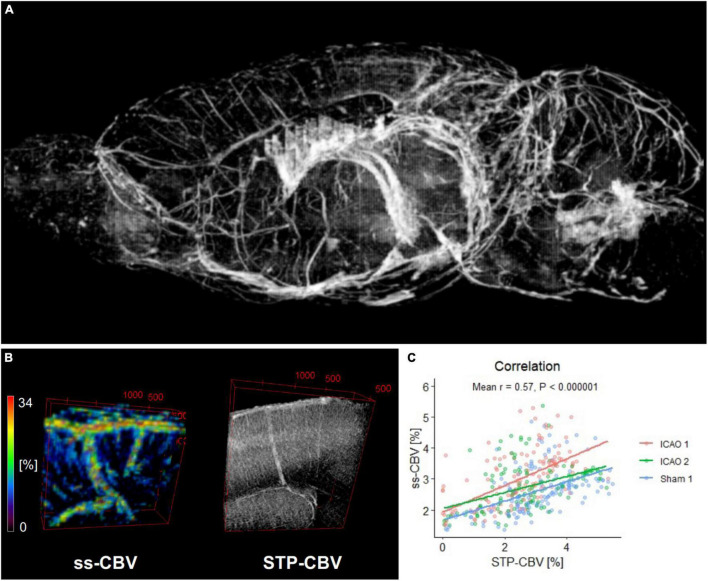
Validation of ss-CBV against 3D serial two-photon tomography (STP). **(A)** Whole-brain histologic angiography: Perfused blood vessels were stained *in vivo* with lectin from Lycopersicon esculentum and measured by STP. The image shows a 3D reconstruction of large- and medium-size blood vessels of the cerebral vascular tree using the ImageJ plugin Volume Viewer (arbitrary units). **(B)** Panel shows corresponding maps of iron oxide nanoparticle-based ss-CBV and STP-CBV (including the capillary bed) reconstructed from STP (unit: volume percent). **(C)** Positive and significant correlation between STP-CBV and ss-CBV among 406 brain regions defined by the Allen murine brain atlas in 3 mice described by the mean Spearman’s rank correlation coefficient.

Next, ss-CBV findings were histologically validated at the whole brain level by correlating ss-CBV to corresponding STP-CBV from STP angiographies. For this purpose, 406 regions of the Allen brain atlas were covered with MRI and STP modalities to permit a direct comparison of ss-CBV against STP-CBV across those regions. Importantly, blood vessels according to ss-CBV were accurately traceable by STP (Figure 4) and a correlation analysis across 406 brain regions from the Allen brain atlas demonstrated good correlation between ss-CBV and STP-CBV in the mouse neocortex (Mean *r* = 0.57; *p* < 0.000001; Sham: *n* = 1; ICAO: *n* = 2; Figure 4).

## Discussion

In this study, we show that iron oxide nanoparticle-based ss-CBV maps serve as a valid surrogate marker for semi-quantitative assessment of cerebral blood vessels and spontaneous collateral outgrowth in the mouse neocortex with higher resolution and improved contrast compared to rCBV determined by DSC: 21 days after permanent ICAO, spontaneous outgrowth of neocortical blood vessels was mirrored by an increase of ss-CBV, which corresponded to confocal model validation and correlated well with vessel density assessed by STP. The significance of this work is that iron oxide nanoparticle-based ss-CBV can help to refine and reduce experimental protocols by serving as a novel surrogate for longitudinal characterization of cerebrovascular remodeling.

The endogenous outgrowth of collaterals represents the most powerful mechanism to cope with chronic cerebral hypoperfusion (Schaper and Scholz, 2003) and a growing body of evidence has shown preclinical effectiveness of therapeutic stimulation of collateral vessel growth in the murine brain (Buschmann et al., 2003; Schneeloch et al., 2004; Schneider et al., 2007; Duelsner et al., 2012; Hecht et al., 2012, 2015; Marushima et al., 2019). Consequently, longitudinal imaging of development, outgrowth, and remodeling of cerebral collaterals in models of health and disease remains of high interest but is also affected by the ongoing effort to replace, reduce, and refine (3Rs) animal experiments (Blakemore et al., 2012). Although the natural dynamics of cerebral collateral outgrowth and remodeling cannot be completely replaced by *ex vivo* modeling, we believe that reliable establishment of non-invasive, high-resolution CE-MRI has meaningful potential for reduction and refinement because CE-MRI facilitates an improved, longitudinal study design and intra-individual analysis with higher statistical power at the benefit of using a non-invasive technology (Nesselroade and Ram, 2004). For assessment of large caliber vessels, this hypothesis is supported by recent findings showing standardized and reliable time-of-flight (TOF) MR angiography for quantitative, longitudinal characterization of collateralization at the level of the Circle of Willis in mice (Foddis et al., 2020; Knauss et al., 2020). For assessment of collateral remodeling at the level of penetrating arterioles, however, the resolution of TOF MR angiography is too low and CE-MRA using a blood pooling agent could be better suited (Klohs et al., 2012). In order to test the feasibility and validity of iron oxide nanoparticle-based CE-MRA for quantification of parenchymal vascular remodeling, we therefore deliberately chose a previously characterized model of unilateral attenuation of cerebral perfusion in mice, in which collateral remodeling not only occurs within the basal and leptomeningeal vasculature, but also within the parenchymal resistance vessels in the neocortex of both hemispheres (Hecht et al., 2012). We clearly observed a higher (micro)vascular density in iron oxide nanoparticle-based CE-MRA subtraction angiograms on Day 21 after chronic hypoperfusion in the right hemisphere as the predefined region of interest that has predominantly been found hemodynamically impaired after right-sided ICAO (Hecht et al., 2012). The vessel density in the left hemisphere contralateral to ICAO also seemed to increase in line with previous findings (Hecht et al., 2012), however, including the left hemisphere as the second region of interest would have underpowered our analysis. However, the observation of increased vessel density in the right hemisphere did *not* translate into a higher vessel density using a previously established semi-automated vessel tracing algorithm. Considering the generally intact blood-brain barrier within the parenchymal vasculature following unilateral ICAO (Hecht et al., 2015), this unexpected finding could be explained by blooming artifacts that confounded the algorithm especially in animals with ICAO and collateral remodeling, because increasing vessel volumes possibly results in an inability of the algorithm to correct for overlapping blood vessels on subtraction angiograms. Thus, semi-automated vessel tracing algorithms for CE-MRA subtraction angiograms may require individual validation depending on the underlying model of disease.

Under the hypothesis that cerebral blood volume maps could serve as a more robust surrogate marker for vessel density assessment, we then calculated ss-CBV with the cylinder model of blood vessels in the static dephasing regime of the MR signal to mitigate the limitation of interfering blooming artifacts (Boxerman et al., 1995). The effect of iron oxide nanoparticle dosage for ss-CBV is largely independent of concentration and the method has proven valid within a large range of concentrations both theoretically (Yablonskiy and Haacke, 1994) and experimentally (Tropres et al., 2004). In the present study, a significant increase in ss-CBV on Day 21 after ICAO was noted. Assuming equal contrast agent concentration and pulse sequence among all measurements, this higher ss-CBV after ICAO most likely represents an intraparenchymal vessel volume increase due to remodeling of the neocortical vasculature in line with our previous observations in the murine neocortex (Hecht et al., 2012, 2015; Marushima et al., 2019). For internal validation, ss-CBV was additionally compared to DSC-rCBV because DSC represents one of the most widely applied techniques for MRI-based CBV estimation. In our present setup, we found that both techniques can easily be combined in one protocol by low resolution fast imaging during bolus injection of the iron oxide nanoparticle (DSC-rCBV) followed by high resolution imaging after the blood pool agent has reached equilibrium concentration in plasma (ss-CBV). Importantly, ss-CBV was paralleled by DSC-rCBV values within limits of agreement but at clearly higher image resolution and contrast, which is explained by the strong effect of iron oxide nanoparticles on T2* shortening, the absence of contrast agent leakage, and the possibility of longer image acquisition time during steady-state blood volume mapping using iron oxide nanoparticles compared to bolus techniques with rapid first-pass effect, such as conventional blood volume mapping based on DSC (Guckel et al., 1994; Bremer et al., 2003; Wu et al., 2003; Varallyay et al., 2013). For definite morphological validation, ss-CBV was then compared to co-registered histology based on 3D whole brain STP. The significant correlation between ss-CBV and histological STP-CBV supported the hypothesis that ss-CBV can serve as a robust surrogate for blood vessel assessment. Importantly, for the first time we provide a systematic morphological validation of iron oxide nanoparticle-based ss-CBV maps by co-registering a comprehensive and corresponding complete histological data set, which is highly relevant considering that our atlas registration approach eliminates selection bias introduced by region-of-interest-based analyses and remains unaffected by inter-rater heterogeneity. Therefore, ss-CBV maps could serve as a method of choice for robust, semi-quantitative, and longitudinal blood vessel assessment at the level of the neocortical vasculature.

Alternative emerging approaches to quantify ferumoxytol enhanced vasculature are susceptibility weighted imaging (SWI) and quantitative susceptibility mapping (QSM), which even enabled us to distinguish between vessel diameters (Liu et al., 2018; Buch et al., 2020; Shen et al., 2020). However, none of the studies performed histological validation and to our best knowledge, no SWI/QSM protocol for the mouse has been published in the context of cerebral microangiography. Furthermore, QSM studies require sophisticated postprocessing, which needs to be fed by complex simulations. Due to robustness, we decided for ss-CBV in this study, since the disadvantage of insensitivity of vessel diameter is an advantage at the same time: the results have no bias toward any vessel diameter. Thus, ss-CBV currently represents a more established parameter also applicable to very small vessels and independent of resolution.

### Limitations

Clinically, iron oxide nanoparticle-based CE-MRA and ss-CBV maps could serve as a non-invasive alternative to digital subtraction angiography for longitudinal assessment of vascular malformations, brain tumor vascularity, anti-angiogenic treatment effects but also for improved planning of neurosurgical or radiosurgical procedures (Varallyay et al., 2013; Iv et al., 2018; Huang et al., 2019). However, clinical application has been limited to proof-of-concept studies and off-label use due to missing regulatory approval, which is mainly attributed to the concern that the carbohydrate coating of the nanoparticles could be associated with an increased risk of severe allergic response. On the other hand, the safety profile of iron oxide nanoparticles has been reported similar to iodinated contrast agents used for contrast-enhanced CT imaging (Vasanawala et al., 2016). Additionally, there is increasing awareness of permanent gadolinium deposition in the brain and its unknown risk (Gulani et al., 2017) and iron-based contrast agents may be considered as a suitable alternative (Boehm-Sturm et al., 2018).

A main limitation in the present study is the sample size. However, we deliberately decided to only use animals from one sex and a single model of disease because we believe that this optimizes the reliability of our findings by reducing the complexity of the model. Second, the lack of coherence to the previously published vessel tracing algorithm for CE-MRA underlines that such approaches may require individual validation depending on the used model or the diversity of the cohort, including the use of subjects of both sexes and different mouse strains. Third, validation of ss-CBV measurements were naturally limited by the fact that our STP vessel stain only reflected blood vessel walls rather than appreciate the entire volume of the vessel lumen as it is done by ss-CBV. On the other hand, the fact that ss-CBV recognizes the entire vessel lumen also explains why this method seems particularly useful for microvascular vessel volume quantification, because histological CBV may underestimate vessel density in areas of large surface vessels (Di Giovanna et al., 2018). Forth, although ss-CBV offers the possibility to determine vessel density based on perfused blood volume with the benefit of being extremely robust, ss-CBV is unable to distinguish between vessel diameters. For this purpose, vessel size imaging and quantitative blood oxygen level dependent imaging make use of more advanced biophysical models of spin echo MR signal in tissue (Kiselev and Novikov, 2018) but are currently limited by high noise level and low spatial resolution (Boehm-Sturm et al., 2013). MR vascular fingerprinting is a promising first attempt to reduce noise by simulating the MR signal in realistic tissue and finding a best match to the measured signal (Lemasson et al., 2016), which may soon enable vessel size sensitive imaging at similarly high resolution presented here while reducing impact of noise. In the future, automated machine-learning analyses of comprehensive histological and MR angiograms may help overcome the limitations of previous and the present work and yield further insights into neurovascular imaging correlates with the possibility of reliable clinical translation (Di Giovanna et al., 2018; Todorov et al., 2019).

## Data Availability Statement

The datasets presented in this study can be found in online repositories. The names of the repository/repositories and accession number(s) can be found below: https://gin.g-node.org/tdebortoli/CE_MRI.git.

## Ethics Statement

The animal study was reviewed and approved by the local ethics committee on animal research (Landesamt für Gesundheit und Soziales, Berlin, Germany; LAGeSo approval number G0186/16).

## Author Contributions

NH and PB-S conceived and designed the study. NH drafted the Ethics committee proposal and supervised the project. TB performed the animal surgeries, tissue preparation for histology, confocal model validation analysis, and drafted the figures. PB-S, SK, and SM designed the MRI protocols. TB, PB-S, and SK performed the MRI validation analysis. TB and NH performed the statistical analysis. TB, PB-S, and NH created the first draft of the manuscript. All authors were involved in analysis and interpretation of the data and contributed to critical revision of the final manuscript.

## Conflict of Interest

The authors declare that the research was conducted in the absence of any commercial or financial relationships that could be construed as a potential conflict of interest.

## Publisher’s Note

All claims expressed in this article are solely those of the authors and do not necessarily represent those of their affiliated organizations, or those of the publisher, the editors and the reviewers. Any product that may be evaluated in this article, or claim that may be made by its manufacturer, is not guaranteed or endorsed by the publisher.

## Footnotes

1
https://imagej.nih.gov/ij/
