# Supplementary material for: Three-Dimensional Iron Oxide Nanoparticle-Based Contrast-Enhanced Magnetic Resonance Imaging for Characterization of Cerebral Arteriogenesis in the Mouse Neocortex
Source: Front Neurosci. 2021 Nov 26;15:756577. doi: 10.3389/fnins.2021.756577 (PMC8662986; doi:10.3389/fnins.2021.756577)
Supplement: Supplementary file 1 [file Data_Sheet_1.pdf]

Supplementary Material

Supplementary Figures

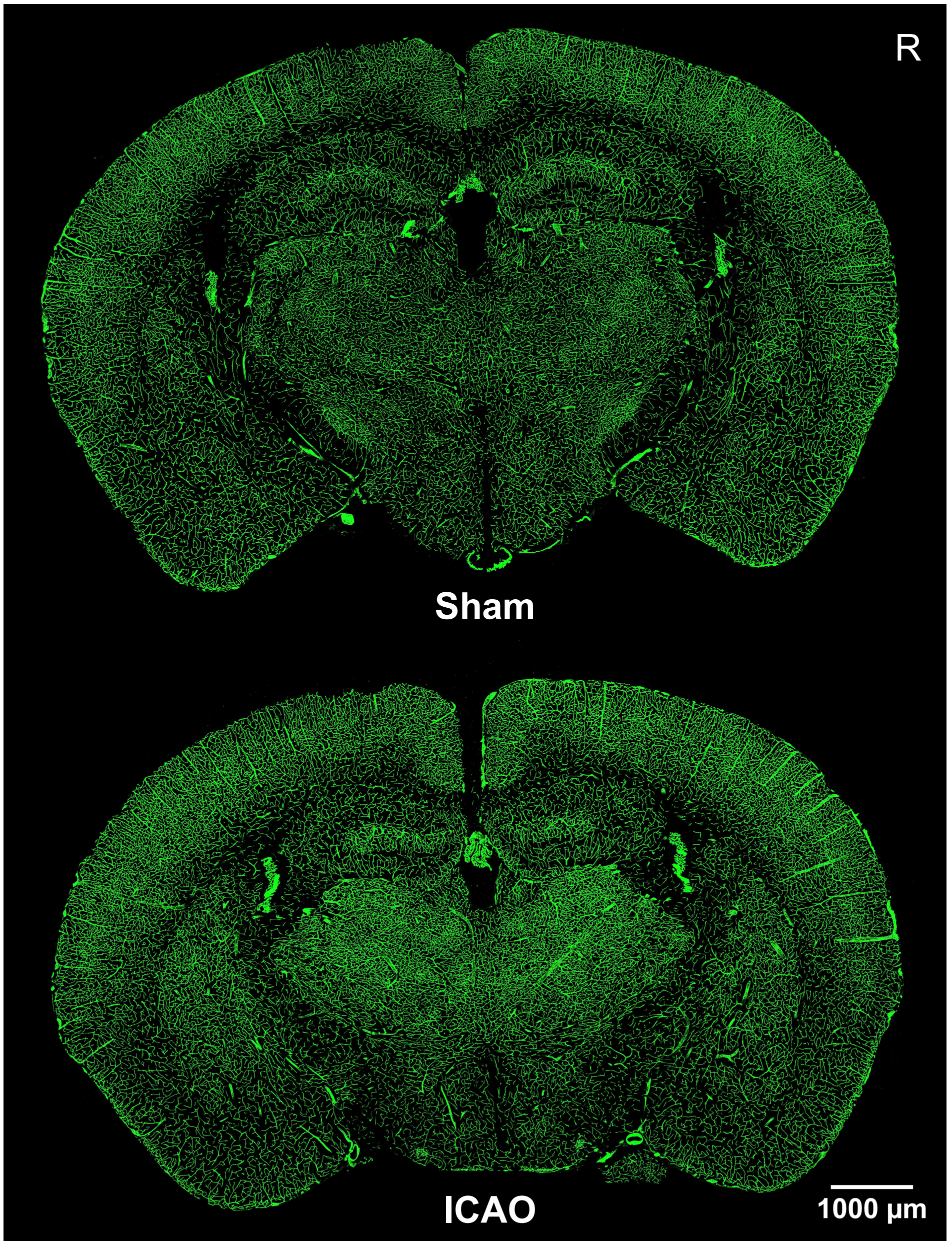

Supplementary Figure 1. *Histology of cerebral vasculature on day 21 following Sham or ICAO.* Lectin-stained perfused blood vessels were visualized by confocal laser scanning microscopy. The images show exemplary maximum intensity projections of image stacks with 80µm depth for Sham (upper image) and ICAO (lower image) for calculation of confocal cerebral blood vessel volume (confocal-CBV) in the right neocortex.

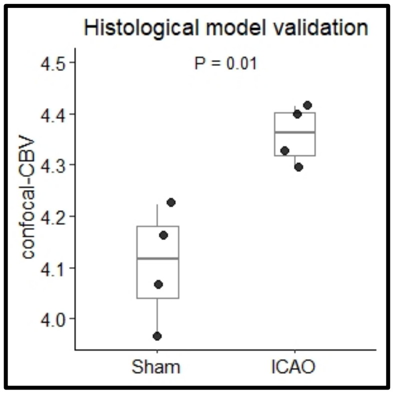

Supplementary Figure 2. *Histological model validation.* A significant increase of the histological vessel volume measured by confocal laser scanning microscopy (confocal-CBV) was found in the right-sided neocortex 21 days after ICAO. *p=0.01 for Sham versus ICAO, two-sided t-test.

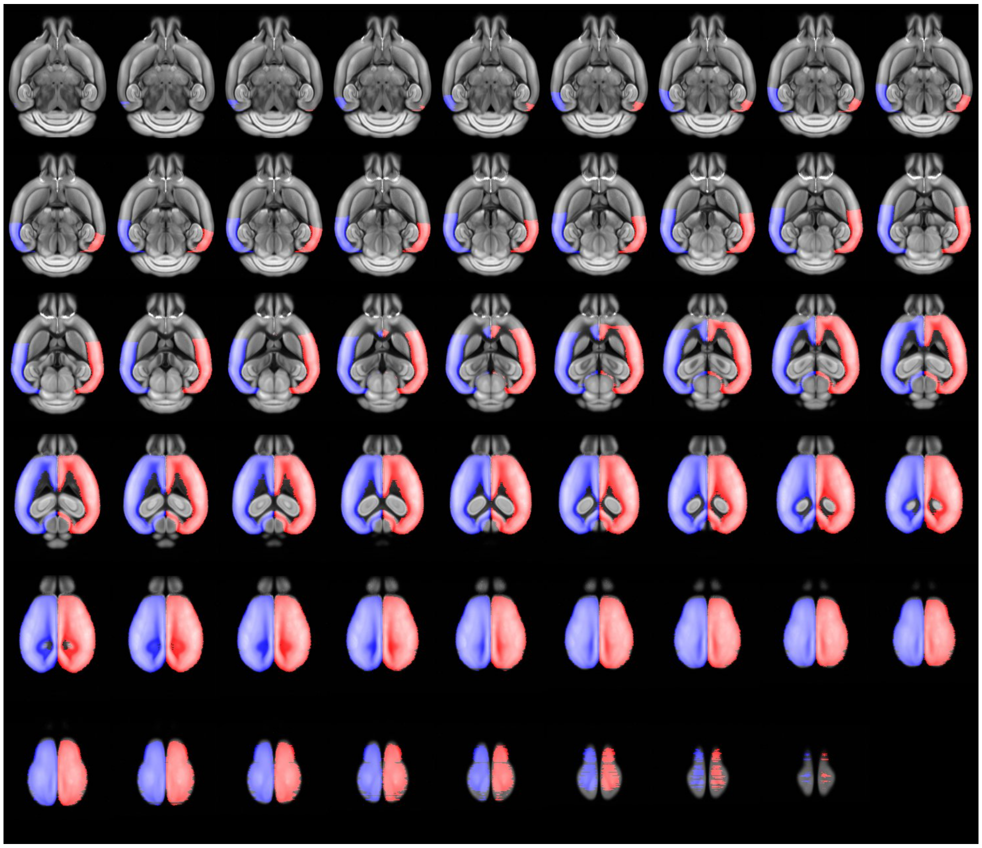

Supplementary Figure 3. *Region of interest (ROI) for ss-CBV analysis.* The red area illustrates the ROI within the right-sided neocortex selected for ss-CBV analysis. The blue area represents the corresponding left-sided neocortex. The grey area shows an average template of a C57BL/6J brain.
